# Relation between Actinomycosis and Histopathological and Clinical Features of the Palatine Tonsils: An Iranian Experience

**Published:** 2011-07-01

**Authors:** M J Ashraf, N Azarpira, B Khademi, B Hashemi, M Shishegar

**Affiliations:** 1 Department of Pathology, Shiraz University of Medical Sciences, Shiraz, Iran; 2 Transplant Research Center, Nemazee Hospital, Shiraz University of Medical Sciences, Shiraz, Iran; 3 Department of Otolaryngology, Shiraz University of Medical Sciences, Shiraz, Iran

**Keywords:** Actinomycosis, Tonsils, Prevalence, Iran

## Abstract

**Background:**

Actinomycosis of the tonsils has been reported in a variable percentage of tonsil specimens by other authors. This study determines the incidence of actinomyces in the palatine tonsil and evaluates the clinical diagnoses and histopathological features of tonsillectomy specimens.

**Methods:**

In a retrospective study, 204 patients who had undergone tonsillectomy for recurrent tonsillitis (group A) and for sleep apnea without a history of recurrent tonsillitis (group B) were enrolled.

**Results:**

The prevalence rate was significantly higher in the adult compared with the pediatric population. The prevalence of tonsillar actinomycetes colonization was higher in patients who had undergone tonsillectomy for recurrent tonsillitis (43.9%) than in patients who had undergone tonsillectomy for obstructive sleep apnea (26.3%). The prevalence did not differ by sex of patient. Histopathological analysis of resected tonsils did not show active tissue infection. There was a statistically significant relationship between the presence of actinomycosis and age, with a greater occurrence of actinomycosis in adult patients.

**Conclusion:**

Although actinomyces colonization is more prevalent in patients with recurrent tonsillitis than sleepdisordered breathing, but the presence of actinomyces does not indicate any active disease.

## Introduction

Actinomyces are slow growing, gram positive, nonacid fast, anaerobic, comensal bacteria within oral cavity, colon and vagina. The most frequently isolated pathogenic Actinomycetes are Actinomyces Israeli and Actinomyces naeslundi. In vivo growth of actinomycetes usually results in the formation of characteristic clumps called grains or sulfur granules. Actinomycosis is not an opportunistic infection and usually occurs in healthy individuals, however an increasing number of reports have described an association with HIV infection, transplantation and radio- or chemotherapy.[1] This infection is commonly seen and reported in craniofacial, ileocecal, pulmonary regions and the vaginal smear of intrauterine device users. It is also reported rarely at other sites such as liver, breast, parotid, muscle, prostate and spleen.[2][3] Penicillin continues to be the standard treatment for actinomycosis.[3] Actinomycosis of the tonsils has been reported in a variable percentage of tonsil specimens by other authors.[3][4][5][6][7] It has been suggested that Actinomycosis infection of the tonsils may indicate an etiological role for this organism in tonsillar and adenoidal hypertrophy.[3][6] Other authors have implicated this organism as merely a saprophyte of the normal tonsil.[5] Its role in disease of the tonsils is therefore not clearly understood. Actinomycetes are anaerobes that release proteolytic enzymes which diminish oxidation-reduction potential and leads to proliferation of the organisms that invades the surrounding tissues.[6] This may lead to the colonisation of the tonsils by the actinomycotic organisms normally present within the tonsillar crypts.[3][6]

Hematoxylin-and-eosin (H&E) staining has been shown to be highly effective in detecting Actinomyces colonies.[5][6] The presence of actinomycosis can be recognized as aggregates of filamentous basophilic microorganisms arranged in a radial spoke-like fashion; the so-called “ray-fungus” appearance of an Actinomyces colony.[3][5][6] The objective of the study is to determine the incidence of actinomycosis in the tonsils of patients undergoing tonsillectomy, and to evaluate its clinical role.

## Materials and Methods

We conducted a retrospective study at the Khalili Hospital affiliated to Shiraz University of Medical Sciences, in Shiraz, Southern Iran between January 2007 and July 2008. Two hundreds and four patients undergoing elective tonsillectomy during this period were included in the study. The following data were collected: age, sex, indication for surgery, presenting symptoms, tonsil size and tonsil asymmetry. Tonsillectomy was performed with dissection or bipolar diathermy. The tonsils were fixed in formalin, embedded in paraffin and a microscopic slide was prepared. The slides were stained with H&E. The assessment of each tissue sample included evaluation of mucosal surface, crypts and associated lymphoid tissue. Actinomycosis was recognised as aggregates of filamentous basophilic microorganisms arranged in a radial spoke-like fashion.[6][8] All slides was reevaluated for presence of cryptitis, active tonsillitis and location of the Actinomyces groups ;eg deep within the tonsillar crypts or only superficially present.

A clinicopathological comparison was made between the patients with and without actinomycosis. Two groups of patients were identified based on the indication for surgery. The group A consisted of those patients operated on for obstructive sleep apnea and group B was operated for recurrent tonsillitis. The incidence of actinomycosis in each of these groups was determined separately. Another comparison was made between the incidence of actinomycosis in patients aged ≤ 20 years and those aged >20 years. The data were statistically analyzed to determine the association between actinomycosis of the tonsils and age, sex, histopathological and clinical diagnosis. The results are expressed as mean±SD, t and Chi-Square tests were used as appropriate. We used SPSS version 15.0 (Chicago, IL, USA) for the analysis of the statistical data. A p value less than 0.05 was considered significant.

## Results

Four hundred tonsils were analysed in 204 patients. The mean age of patients was 18.93±10.36 years (range 3-72 years). There were 78 males and 126 females. We found 83 patients (40.7%) with actinomycosis in the tonsils. The clinicopathological features of these patients are summarized in Table 1. The mean age of patients with actinomycosis was 23.34 years and without the disease was 15.91 years. There was a statistically significant relationship between the presence of actinomycosis and older age (p<0.001). There was no pertinent sex-related difference in incidence.

The most common indication for surgery was recurrent tonsillitis in 166 patients (81%) and obstructive sleep apnea in 38 patients (19%) (Table 2). There were 38 patients in group A (obstructive sleep apnea) and of these 10 patients (26.3%) had actinomycosis (Table 2). In group B (recurrent tonsillitis), 43.9% of patients (73/166 patients) had actinomycosis. The difference in incidence of actinomycosis between these two groups was not statistically significant.

**Fig. 1A and 1B:**
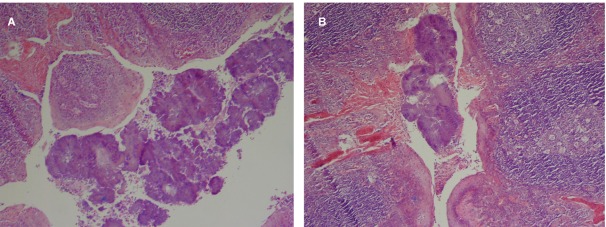
Actinomycosis within the tonsillar crypt (H and E staining ×400)

The histology results of all the specimens examined revealed that reactive follicular hyperplasia was present in all (100%) patients. Actinomycosis was associated with reactive follicular hyperplasia Fig 1A and 1B. No specific tissue reaction to Actinomyces was seen and there was only tonsillar colonization. In 30 patients, the Actinomycetes colonies were seen deep within the tonsillar crypts and in 53 patients they were situated superficially.

**Table 1:** Comparison in clinicopathological findings between patients with actinomycosis of the tonsils and without actinomycosis

**Clinical findings**	**With Actinomycosis**	**Without Actinomycosis**	***P* value**
Total	83	121	
Sex (M/F)	32/51	46 / 75	0.527
Average age	23.34±(10.02 )	15.91±(9.51 )	<0.001
Age≤20	21 (20.4%)	82 (79.6%)	
Age>20	62 (61.4%)	39 (38.6%)	<0.001

**Table 2:** Incidence of actinomycosis according to indication of surgery and age grouping

**Group**	**Total**	**Number of patients with actinomycosis (%)**	***P* value**
Group A (Obstructive sleep apnea )	38	10 (26.3)	0.168
Group B (Recurrent tonsillitis )	166	73 (43.9)	
Age≤20	103	21 (20.8)	0.168
Age>20	101	62 (61.38)	

## Discussion

The presence of actinomycosis in the tonsils has been identified in 1896. In the more recent studies, the occurrence rates seen are between 2-30%.[3][5][6][10][11][12] Our study had a higher incidence (40.6%). The causes of this were different sectioning and staining techniques employed by different laboratories, variations in the groups of patients studied, and variations in the indications for tonsillectomy.[3][5]

A number of series have been published to try and establish the relationship between tonsillar actinomycosis and clinical tonsillar disease.[3][4][5][6][7] Pransky et al.[6] found that histological examination of the core tissues of the tonsils will accurately detect the presence of these organisms.[6] Pransky et al. found an increased prevalence of actinomycosis in patients undergoing adenotonsillectomy6 and postulated that core tissue colonization may be associated with lymphoid hyperplasia and obstructive symptoms. They suggested an initial 12-week course of oral penicillin be administered to all patients with obstructive symptoms to eradicate the organism and diminish the tonsillar size. Bhargava et al. similarly found a positive correlation between actinomycosis and tonsillar hypertrophy; actinomycosis was present in 56.8% of patients with tonsillar hypertrophy compared to 10.3% of patients with recurrent tonsillitis.[3] They also found association of tonsillar actinomycosis with sickle cell anemia, beta thalassemia, bronchial asthma and beta hemolytic Streptococcal infections and suggested tonsillectomy as the treatment of choice for tonsillar actinomycosis.[3]

Gaffney et al. studied the association between recurrent tonsillitis and actinomycosis and found no increase in the incidence of infection in patients with recurrent tonsillitis compared to a control group.[5] Two other series found no significant association between the presence of tonsillar actinomycosis and tonsillar disease. Aydin et al. in a series of 1820 tonsillectomies found the incidence of actinomycosis to be 6.7% and found no correlation between the clinical diagnosis of tonsillar disease and the presence of actinomycosis.[4] Melgarejo et al.[7] similarly found no relationship between Actinomyces and recurrent tonsillitis or adenotonsillar hypertrophy. The present study found no significant relationship between the presence of actinomycosis and tonsillar hypertrophy and/or recurrent tonsillitis (p=0.85).

The histological examination of the tonsils of the 83 patients with tonsillar actinomycosis in the present study revealed no specific tissue reaction to Actinomyces in any of the specimens. There was only colonization of the tonsils. Gaffney et al. reviewed 42 tonsils with actinomycosis and found no correlation between the presence of actinomycosis and tonsillar fibrosis or micro-abscesses, and concluded that actinomycosis was present only as a saprophyte.[5] Aydin et al. found a significantly higher rate of cryptitis in tonsils with actinomycosis and suggested that cryptitis be used as a histopathologic indicator for tonsillar actinomycosis.[4]

Our study showed correlation between actinomycosis and increasing age (p<0.001). Malgerejo et al. reported that the incidence of actinomycosis was more prevalent in patients aged 5- 16 years.[7] Aydin et al. and Toh et al., also found actinomycosis more common in adults than in children.[4][13] It does therefore seem that actinomycosis of the tonsils is associated more commonly with older children and adults.

In conclusion, no correlation was found between the presence of tonsillar actinomycosis and recurrent tonsillitis and/or obstructive tonsillar hypertrophy. Histopathologic findings showed no evidence of tissue reaction to Actinomyces and its presence was found only due to colonization of the tonsils. There was significant correlation between actinomycosis colonization and age that actinomycosis being more common in older patients.

## References

[1] van Lierop AC, Prescott CA, Sinclair-Smith CC (2007). An investigation of the significance of Actinomycosis in tonsil disease. Int J Pediatr Otorhinolaryngol.

[2] Azarpira N, Ghasemzadeh B (2005). Splenic Actinomycosis. IJMS.

[3] Bhargava D, Bhusnurmath B, Sundaram KR, Raman R, Al Okbi HM, Al Abri R, Date A (2001). Tonsillar actinomycosis: a clinicopathological study. Acta Trop.

[4] Aydin A, Erkiliç S, Bayazit YA, Koçer NE, Ozer E, Kanlikama M (2005). Relation between actinomycosis and histopathological and clinical features of the palatine tonsils: a comparative study between adult and pediatric patients. Rev Laryngol Otol Rhinol (Bord).

[5] Gaffney R, Harrison M, Walsh M, Sweeney E, Cafferkey M (1993). The incidence and role of actinomyces in recurrent acute tonsillitis. Clin Otolaryngol Allied Sci.

[6] Pransky SM, Feldman JI, Kearns DB, Seid AB, Billman GF (1991). Actinomycosis in obstructive tonsillar hypertrophy and recurrent tonsillitis. Arch Otolaryngol Head Neck Surg.

[7] Melgarejo Moreno P, Hellin Meseguer D, Marco Garrido A, Galindo Ortego X, Ruiz Macia JA, Hostalet F (2006). A correlation between age and Actinomyces in the adenotonsillar tissue in children.. B-ENT.

[8] Brook I, Yocum P, Shah K (1980). Surface vs core tonsillar aerobic and anaerobic flora in recurrent tonsillitis. JAMA.

[9] Slack J (1942). The source of infection in actinomycosis. J Bacteriol.

[10] Maher A, Bassiouny A, Bucci TJ, Moawad MK, Hendawy DS (1982). Tonsillomycosis: a myco-histopathological study. J Laryngol Otol.

[11] Martins RH, Heshiki Z, Luchesi NR, Marques ME (1991). Actinomycosis and botryomycosis of the tonsil. Auris Nasus Larynx.

[12] Assimakopoulos D, Vafiadis M, Askitis P, Sivridis E, Skevas A (1992). The incidence of Actinomyces israeli colonization in tonsillar tissue. A histopathological study. Rev Stomatol Chir Maxillofac.

[13] Toh ST, Yuen HW, Goh YH (2007). Actinomycetes colonization of tonsils: a comparative study between patients with and without recurrent tonsillitis. J Laryngol Otol.

